# Patient characteristics and predictors of mortality among children hospitalised with tuberculosis: A six-year case series study in Uganda

**DOI:** 10.1371/journal.pone.0301107

**Published:** 2024-05-28

**Authors:** Pauline Mary Amuge, Greta Lassance Becker, Rogers Nelson Ssebunya, Esther Nalumansi, Alex Adaku, Michael Juma, Jay Brooks Jackson, Adeodata Rukyarekere Kekitiinwa, Peter James Elyanu, Eric Wobudeya, Robert Blount

**Affiliations:** 1 Research Department, Baylor College of Medicine Children’s Foundation-Uganda, Kampala, Uganda; 2 Division of Pulmonary and Critical Care Medicine, Carver College of Medicine, University of Iowa, Iowa City, Iowa, United States of America; 3 Department of Medical Records, Mulago National Referral Hospital, Kampala, Uganda; 4 Fort Portal Regional Referral Hospital, Kabarole District, Fort Portal City, Uganda; 5 Department of Pathology, University of Iowa, Iowa City, Iowa, United States of America; 6 Department of Paediatrics & Child Health, Mulago National Referral Hospital, Kampala, Uganda; Stellenbosch University, SOUTH AFRICA

## Abstract

**Background:**

The high case-fatality rates among children with tuberculosis (TB) are reportedly driven by in-hospital mortality and severe forms of TB. Therefore, there is need to better understand the predictors of mortality among children hospitalised with TB. We examined the patient clinical profiles, length of hospital stay from date of admission to date of final admission outcome, and predictors of mortality among children hospitalised with TB at two tertiary hospitals in Uganda.

**Methods:**

We conducted a case-series study of children below 15 years of age hospitalised with TB, from January 1^st^, 2016, to December 31^st^, 2021. Convenience sampling was done to select TB cases from paper-based medical records at Mulago National Referral Hospital (MNRH) in urban Kampala, and Fort Portal Regional Referral Hospital (FRRH) in rural Fort Portal. We fitted linear and logistic regression models with length of stay and in-hospital mortality as key outcomes.

**Results:**

Out of the 201 children hospitalised with TB, 50 were at FRRH, and 151 at MNRH. The male to female ratio was 1.5 with median age of 2.6 years (Interquartile range-IQR 1–6). There was a high prevalence of HIV (67/171, 39%), severe malnutrition reported as weight-for-age Z-score <-3SD (51/168, 30%). Among children with pulmonary TB who initiated anti-tuberculosis therapy (ATT) either during hospitalisation or within seven days prior to hospitalisation; cough (134/143, 94%), fever (111/143, 78%), and dyspnoea (78/143, 55%) were common symptoms. Children with TB meningitis commonly presented with fever (17/24, 71%), convulsions (14/24 58%), and cough (13/24, 54%). The median length of hospital stay was 8 days (IQR 5–15). Of the 199 children with known in-hospital outcomes, 34 (17.1%) died during hospitalisation. TB meningitis was associated with in-hospital mortality (aOR = 3.50, 95% CI = 1.10–11.17, p = 0.035), while male sex was associated with reduced mortality (aOR = 0.33, 95% CI = 0.12–0.95, p = 0.035). Hospitalisation in the urban hospital predicted a 0.48-day increase in natural log-transformed length of hospital stay (ln-length of stay) (95% CI 0.15–0.82, p = 0.005), but not age, sex, HIV, malnutrition, or TB meningitis.

**Conclusions:**

In-hospital mortality was high, and significantly driven almost four times higher by TB meningitis, with longer hospital stay among children in urban hospitals. The high in-hospital mortality and long hospital stay may be reduced by timely TB diagnosis and treatment initiation among children.

## Introduction

Among the 10.6 million people with TB globally in 2021 [1], 14% were HIV-negative children and 11% were children living with HIV [1]. Following exposure to TB, children progress from TB infection to TB disease within the first year after exposure, with TB-related mortality as high as 50% [2]. Despite the increasing access to TB preventive treatment in recent years, many children progress to TB disease due to prevailing risk factors like, malnutrition, human immunodeficiency virus (HIV) infection, age below 2 years, severe immune suppression, missed BCG vaccination, and being in close contact with sputum-positive TB patients [3, 4].

TB diagnosis in children is often challenging due to limited options of point-of-care diagnostic tests and difficulty obtaining biological samples [5]. The paucibacillary TB disease in children also makes bacteriological confirmation of TB challenging. Additionally, TB screening and diagnosis is not commonly integrated in routine clinical care algorithms of common childhood illnesses [6–8]. These challenges delay TB diagnosis and treatment initiation in children, contributing to rapid progression to severe disease and increasing the risk of TB-associated mortality [5, 6]. Among children and adolescents living with HIV, at least one out of ten hospitalisations are due to TB [9–11]. However, there is limited documentation of the average length of hospital stay among children hospitalised with TB, and whether the duration of hospital stay significantly predicts in-hospital mortality for the children living with HIV, or those who are HIV negative.

In Uganda, a high TB-HIV burden country, 12% of the annual TB cases are children 0–14 years of age [12, 13]. Children have an average of 6.7 healthcare encounters before making a TB diagnosis, faced with high travel costs to cover long distances [12, 14]. Eventually, these children arrive very ill at the tertiary health facilities, with severe forms of TB disease, resulting in high in-hospital mortality [11]. Previously reported risk factors for death among TB patients in Uganda include; age above 65 years, ≥2km distance from the treating health facility, HIV infection, and lack of bacteriological TB confirmation [15]. Among children treated for TB during six years at an out-patient paediatric TB clinic in Mulago Hospital in Uganda, overall mortality was 7%, with history of hospitalisation being a risk factor for mortality [16]. Most of the available data describes children treated for TB within out-patient settings, with limited data on patient characteristics and risk factors for in-hospital mortality among children hospitalised with TB in Uganda. We opted for a case-series study design employing convenient sampling methodology, rather than a retrospective cohort, primarily due to constraints on accessing data across the entire population over the specified period. The case-series study design enable us to simultaneously study the exposure as hospitalisation with TB, and the key outcome as mortality which complements available evidence from cohorts.

Given the high burden of paediatric TB, our objective was to describe hospitalised patient clinical profiles including length of hospital stay, evaluate the predictors of in-hospital mortality, and determine length of hospital stay among children hospitalised with TB at two referral hospitals in Uganda from 2016–2021.

## Methods

### Study design and study population

We conducted a case-series study during the study period January 2022 to January 2023, among children were hospitalised at two referral hospitals from January 2016-December 2021. Children included were age 0–14 years, with bacteriologically confirmed or clinical TB disease diagnosed by clinicians. TB diagnosis and treatment were conducted based on the National TB and Leprosy Program (NTLP) guidelines [17]. We included children with pulmonary and extra-pulmonary TB (EPTB) who were either new or retreatment patients, with both bacteriologically confirmed or clinically diagnosed TB.

### Study setting

This study was conducted at Mulago National Referral Hospital (MNRH) in urban central Uganda, and Fort Portal Regional Referral Hospital (FRRH) in rural Western Uganda. Fort Portal Regional Referral Hospital serves eight districts in the Rwenzori mountain region of Western Uganda, with an estimated catchment of 3.0 million people. Fort Portal Regional Referral Hospital runs a daily TB clinic for both children, adolescents, and adults. Mulago National Referral Hospital serves as the referral hospital for the city of Kampala’s 1.6 million residents, as well as serving as the national referral specialised hospital for patients from the entire country with complex and severe diseases. Mulago hospital runs a paediatric and adolescent TB clinic, separate from the adult TB clinics. Fort Portal Regional Referral Hospital runs one TB clinic that attends to all children, adolescents, and adults. Access to specialised care and diagnostics are similar at both hospitals.

Children presenting with TB symptoms at both hospitals are usually screened for TB using the WHO-recommended four-question symptom screening guide. Children with presumptive TB undergo comprehensive clinical evaluation and investigations to confirm or rule-out TB. According to the national TB management and control guidelines [17], free Ziehl Neelsen (ZN) microscopy or GeneXpert testing is done on biological samples if successfully collected by the health workers such as expectorated sputum, gastric aspirate/lavage, naso-pharyngeal aspirate, induced sputum, or lymph node aspirate/biopsy. Samples for extra-pulmonary TB (EPTB) diagnosis are collected and tested based on the clinical presentation and suspected location of EPTB. Chest X-ray services were available at both sites. Chest X-rays were also done for some patients who were able to afford it. Sputum culture and sensitivity is the gold standard for TB diagnosis. Sputum samples were referred to the national TB reference laboratory (NTRL) for TB culture and sensitivity if this was requested by the attending clinicians. Health workers follow the approved algorithm for diagnosis of TB in children, which provides guidance on making a diagnosis of bacteriologically confirmed TB (P-BC) in children with positive microbiological test results. However, a negative TB test does not rule-out TB disease in children. Therefore, the diagnostic algorithm provides guidance on clinically diagnosed pulmonary TB (P-CD) in children with negative microbiological test results who have highly suggestive TB symptoms, signs, history of TB contact and suggestive chest X-ray film. Children who are very ill are hospitalised, for collection of biological samples to conduct TB diagnostic tests and to receive treatment. Children diagnosed with TB are initiated on drug-susceptible TB treatment. Hospitalised children are managed by a multi-disciplinary team composed of nurses, doctors, and paediatricians. The national TB management and control guidelines recommend TB treatment adherence to be supported as either directly observed treatment (DOTs) within the health facility for hospitalised/inpatient patients, or within the community/home for stable patients who do not require hospitalisation. Patients with danger signs and severe disease such as TB meningitis and severe pneumonia with signs of respiratory distress are hospitalised for inpatient care. Stable patients are managed in out-patient clinics.

### Sample size

Sample size calculations to determine the appropriate number of retrospective charts to review were based on the hypothesis that length of hospital stay in days (LOS) for children with TBM is longer in rural vs urban settings. We used the t-statistic as calculated at quesgen.com. We tested associations between hospital setting and outcomes (LOS and mortality) in the entire cohort of children with active tuberculosis. Testing our hypothesis, n = 100, we were adequately powered at >80% to detect differences in LOS outcome of >16 days, an adequate effect size as LOS for children with active TB was typically several weeks (median 36 days, IQR 21–60 days in the [18] cohort.

### Data collection, sampling, and materials

Patient data was accessed from 26^th^ January 2022 to 20^th^ January 2023 after ethical approval of the study protocol. Patients included in the study were selected using convenience sampling. Participant hospital files with individual patient identifiers and clinical data were reviewed by trained study clinicians (nurses and doctors) to confirm eligibility and diagnosis of TB from available clinical notes. Instead of individual names, the eligible participants were assigned unique study identifiers. Trained study staff accessed individual identifying data during data collection and data cleaning, after which names were excluded during data entry into the database for data analysis. All data in the database was anonymized for access by authors. Abstracted data included age, sex, documented HIV status, weight, symptoms at admission, clinical impression at admission and at discharge, date of admission and discharge, and TB treatment start date. HIV testing was not done as part of this study. Patients’ clinical status at discharge was categorised as: died, improved, no change, self-discharge, and referred out. Length of hospital stay (LOS) was reported as the number of days spent in hospital from the date of admission to the date of final clinical status.

Moderate and severe malnutrition were defined using weight-for-age (underweight) Z-score less than two and three standard deviations, respectively [19].

### Statistics and data analysis

To examine the difference in demographic and clinical characteristics by age groups, we used Chi-Squared Tests for categorical variables, analysis of variance (ANOVA) tests for normally distributed continuous variables and Kruskal-Wallis tests for continuous variables with non-normal distributions.

We assessed predictors of mortality (primary outcome) using multivariable logistic regression with the following predictors: age, sex, HIV, weight-for-age Z-score, TB meningitis (TBM), referral hospital site, and days spent waiting treatment initiation. In a subgroup multivariable logistic regression analysis, limited to children who initiated TB treatment during admission, we evaluated predictors of in-hospital mortality, with age, sex, days to TB treatment initiation, and prompt TB diagnosis (using the clinical impression on the day of admission) as predictors. As a secondary outcome, we assessed predictors of length of hospital stay using linear regression with log-transformed length of stay as the outcome variable and tested age, sex, HIV, weight-forage Z-score, and referral hospital site as predictors. We log-transformed the outcome variable to accommodate linear regression model assumptions. To addresses missingness for HIV and malnutrition data, we performed sensitivity analyses on baseline characteristics and primary outcomes comparing 1) those with known HIV status versus missing, and 2) those with known nutritional status versus missing. All analyses were completed using Stata 15SE (Stata Corp, College Station, TX, USA).

### Ethics statement

This study was approved by the Mulago Hospital Research and Ethics Committee (protocol number MHREC 2194), the Uganda National Council for Science and Technology (UNCST), and the University of Iowa Institutional Review Board (IRB number: 202111437). Administrative clearance was also obtained from the directors of Fort Portal Regional Referral Hospital and Mulago national referral Hospital. A waiver of written participant informed consent was granted by the Mulago Hospital Research and Ethics Committee, because historical patient records were used to obtain required data, without interacting with the patients for data collection.

## Results

A total of 201 children hospitalised and diagnosed with TB were included in our analysis, with 151/201 (75%) from the urban referral hospital and 50/201 (25%) from the rural referral hospital. The demographics and TB-related clinical characteristics of the children and adolescents are listed for each age category in Table 1. The median age at hospital admission was 2.6 years (Interquartile range (IQR) 1–6 years). More children with TB were male (119/198, 60%). Of 171 children with documented HIV testing, 67 children (39%) were living with HIV. More than 50% of the children were 2 years and below (51.5%), and 15.7% were young adolescents 10–14 years (Table 1). The prevalence of moderate and severe malnutrition was 38/168 (23%) and 51/168 (30%), respectively. The urban referral hospital exhibited a higher prevalence of severe malnutrition 45/109 (41%) compared to 13/46 (28%) at the rural referral hospital (p = 0.043). However, the overall prevalence of malnutrition (moderate and severe) was similar at both sites (p = 0.49) (data not shown). A four-drug TB treatment regimen (rifampicin, isoniazid, pyrazinamide, and ethambutol) was initiated for 199/201 (99%) children. Among 157 children who initiated TB treatment during their hospitalisation, the median time to initiation was 4 days (IQR 1–7) following hospital admission. Among 196 children with known hospital outcomes, 142/196 (72%) children improved, 34/196 (17%) children died, 12/196 (6.1%) children self-discharged, 6/196 (3.1%) children were transferred to another hospital, and 2/196 (1.0%) children had no change in symptoms on discharge. The median length of hospital stay was 8 days (IQR 5–15) (Table 1).

**Table 1:** Demographics and clinical characteristics by age, n = 201.

	Total	Infants	1–2 years	3–5 years	6–9 years	10–14 years	P value^‡^
	n = 201	n = 46	n = 57	n = 38	n = 29	n = 31	
Sex, n (%)^†^	198 (100)	45 (100)	57 (100)	37 (100)	28 (100)	31 (100)	0.39
Female	79 (40)	17 (38)	21 (37)	12 (32)	12 (43)	17 (55)	
Male	119 (60)	28 (62)	36 (63)	25 (68)	16 (57)	14 (45)	
Referral hospital site, n (%)	201 (100)	46 (100)	57 (100)	38 (100)	29 (100)	31 (100)	0.054
Rural	50 (25)	4 (8.7)	15 (26)	13 (34)	8 (28)	10 (32)	
Urban	151 (75)	42 (91)	42 (74)	25 (66)	21 (72)	21 (68)	
HIV, n (%)	171 (100)	34 (100)	45 (100)	33 (100)	28 (100)	31 (100)	0.36
Negative	104 (61)	16 (47)	31 (69)	21 (64)	16 (57)	20 (65)	
Positive	67 (39)	18 (53)	14 (31)	12 (36)	12 (43)	11 (35)	
Malnutrition*, n (%)	168 (100)	37 (100)	51 (100)	33 (100)	23 (100)	24 (100)	0.45
None	79 (47)	19 (51)	22 (43)	16 (48)	11 (48)	11 (46)	
Moderate	38 (23)	5 (14)	16 (31)	4 (12)	7 (30)	6 (25)	
Severe	51 (30)	13 (35)	13 (25)	13 (39)	5 (22)	7 (29)	
Started ATT during hospitalisation, n (%)	201 (100)	46 (100)	56 (100)	37 (100)	29 (100)	31 (100)	0.66
No	2 (1.0)	0	1 (1.8)	1 (2.6)	0	0	
Yes	199 (99)	46 (100)	56 (98)	37 (97)	29 (100)	31 (100)	
Time to ATT initiation, observations	157	34	50	25	25	23	
Days, median (IQR)	4 (1–7)	4.5 (2–9)	3 (1–7)	5 (2–7)	3 (1–8)	3 (1–6)	0.85
Hospital outcomes, n (%)	196 (100)	45 (100)	56 (100)	37 (100)	28 (100)	30 (100)	0.23
Died	34 (17)	9 (20)	9 (16)	5 (14)	3 (11)	8 (27)	0.51
Improved	142 (72)	32 (71)	42 (75)	28 (76)	21 (75)	19 (63)	
No change	2 (1.0)	2 (4.4)	0	0	0	0	
Self-discharge	12 (6.1)	2 (4.4)	4 (7.1)	4 (11)	1 (3.6)	1 (3.3)	
Referred out	6 (3.1)	0	1 (1.8)	0	3 (10.7)	2 (6.7)	
Length of hospitalisation, observations	193	46	53	36	28	30	
Days, median (IQR)	8 (5–15)	9.5 (5–19)	7 (4–13)	7 (5–14.5)	8 (5–15.5)	11.5 (6–24)	0.41

*Abbreviations*: ATT = anti-tuberculosis therapy; Interquartile range = IQR; HIV = human immunodeficiency virus.

^†^% represents percent column total per characteristic (e.g., sex, hospital site, HIV).

^‡^Determined p-value for comparison between age groups using chi-squared test for categorical variables and *Kruskal*-*Wallis test for continuous* variables.

*Malnutrition is defined as underweight using weight-for-age Z-score.

Pulmonary TB was diagnosed in 153/201 (76%) children and EPTB in 48/201 (24%) children (Table 2). Of the children diagnosed with EPTB, 29/48 (60%) children had TB meningitis, 5/48 (10%) TB lymphadenitis, 4/48 (8.3%) abdominal TB, 7/48 (15%) disseminated TB [3 miliary], and 3/48 (6.3%) cardiac TB. Severe forms of TB were reported among 19% of hospitalised children (TB meningitis, disseminated TB, TB pericarditis).

**Table 2:** Site of tuberculosis disease by age.

Site of disease	Total	Infant	1–2 years	3–5 years	6–9 years	10–14 years	P value
	n = 201	n = 46	n = 57	n = 38	n = 29	n = 31	
	n (%)	n (%)	n (%)	n (%)	n (%)	n (%)	
Pulmonary	153 (76)	37 (80)	47 (82)	28 (74)	22 (76)	19 (61)	0.23
Meningitis	29 (14)	7 (15)	5 (8.8)	6 (16)	4 (14)	7 (23)	0.52
Disseminated	7 (3.5)	1 (2.2)	2 (3.6)	1 (2.6)	1 (3.5)	2 (6.4)	0.89
Cardiac	3 (1.5)	0	0	2 (5.3)	0	1 (3.2)	0.177
Lymphadenitis	5 (2.5)	1 (2.2)	2 (3.5)	1 (2.6)	1 (3.5)	0	0.88
Abdominal	4 (2.0)	0	1 (1.8)	0	1 (3.5)	2 (6.5)	0.27

Among the children who initiated TB treatment either during hospitalisation or within seven days prior to hospitalisation, cough 134/143 (94%), fever 111/143 (78%), and dyspnoea 78/143 (55%) were the common admission symptoms among children with pulmonary TB (Table 3). Children with TBM were admitted with fever 17/24 (71%), convulsions 14/24 (58%), and cough 13/24 (54%). Symptoms of pulmonary TB did not significantly vary by age group. Among children with TBM, convulsions (p = 0.019) and cough (p = 0.017) were less common among children aged 6–9 years than adolescents 10–14 years and young children less than five years.

**Table 3:** Admission symptoms of pulmonary and meningeal tuberculosis by age.

	Total	Infant	1–2 years	3–5 years	6–9 years	10–14 years	P value
	n (%) ^†^	n (%)	n (%)	n (%)	n (%)	n (%)	
**Pulmonary tuberculosis**	n = 143^‡^	n = 34	n = 44	n = 25	n = 21	n = 19	
Cough	134 (94)	32 (94)	42 (95)	24 (96)	19 (90)	17 (89)	0.84
Fever	111 (78)	24 (71)	36 (82)	18 (72)	17 (81)	16 (84)	0.65
Dyspnoea	78 (55)	24 (71)	25 (57)	11 (44)	8 (38)	10 (53)	0.13
Weight loss	38 (27)	5 (15)	10 (23)	10 (40)	5 (24)	8 (42)	0.110
Poor appetite	26 (18)	3 (8.8)	11 (25)	3 (12)	5 (24)	4 (21)	0.33
Diarrhea	26 (18)	7 (21)	12 (27)	2 (8.0)	3 (14)	2 (11)	0.26
Night sweats	21 (15)	1 (2.9)	6 (14)	6 (24)	5 (24)	3 (16)	0.14
Failure to thrive	4 (2.8)	1 (2.9)	1 (2.3)	0	1 (4.8)	1 (5.3)	0.83
**Tuberculosis meningitis**	n = 24^‡^	n = 6	n = 5	n = 3	n = 4	n = 6	
Fever	17 (71)	4 (67)	4 (80)	1 (33)	3 (75)	5 (83)	0.56
Convulsions	14 (58)	5 (83)	4 (80)	3 (100)	0	2 (33)	0.019
Cough	13 (54)	5 (83)	4 (80)	0	0	4 (67)	0.017
Dyspnoea	9 (38)	4 (67)	2 (40)	0	1 (25)	2 (33)	0.37
Weight loss	7 (29)	0	1 (20)	1 (33)	1 (25)	4 (67)	0.146
Night sweats	6 (25)	2 (33)	0	0	1 (25)	3 (50)	0.30
Vomiting	4 (17)	0	2 (40)	0	1 (25)	1 (17)	0.41
Vision changes	2 (8.3)	0	0	0	2 (50)	0	0.028
Fatigue	2 (8.3)	1 (17)	0	0	0	1 (17)	0.70

^†^% represents the number of children with a symptom divided by the total number of children with either pulmonary tuberculosis (n = 143) or tuberculosis meningitis (n = 24).

^‡^Included only children who initiated anti-tuberculosis therapy during hospitalisation or within one week prior to hospital admission.

In unadjusted logistic regression models, predictors of in-hospital mortality included TBM (OR = 3.91, 95% CI = 1.64–9.32, p = 0.002) and hospitalisation in an urban referral hospital (OR = 6.56, 95% CI = 1.51–28.48, p = 0.012) (Table 4). In the adjusted model, male children with TB had 67% reduced odds of dying in the hospital compared to female children (OR = 0.33, 95% CI 0.12–0.95, p = 0.040); while patients with TB meningitis were at 3.5-fold increased odds of mortality compared to patients with other TB sites of disease (aOR = 3.50, 95% CI = 1.10–11.17, p = 0.035).

**Table 4:** Predictors of in-hospital mortality among children with tuberculosis.

	Unadjusted			Adjusted^‡^ (n = 146)		
	OR	95% CI	p-value	OR	95% CI	P value
Age in years (n = 199)	1.02	0.92–1.12	0.75	0.98	0.86–1.11	0.74
Sex (n = 196)						
Female	1			1		
Male	0.83	0.39–1.75	0.62	0.33	0.12–0.95	0.040
Referral hospital site (n = 199)						
Rural	1			1		
Urban	6.56	1.51–28.48	0.012	4.54	0.96–21.53	0.057
HIV (n = 171)						
Negative	1			1		
Positive	1.23	0.55–2.74	0.61	0.50	0.16–1.56	0.23
Weight-for-age Z-score (n = 166)	0.90	0.76–1.08	0.26	0.87	0.71–1.07	0.182
TB meningitis (n = 199)						
No	1			1		
Yes	3.91	1.64–9.32	0.002	3.50	1.10–11.17	0.035

*Abbreviations*: CI = confidence interval; HIV = human immunodeficiency virus; OR = odds ratio; TB = tuberculosis.

^‡^Adjusted logistic regression model, including all variables in the above table.

We found no statistically significant associations between mortality and age, HIV, referral hospital site, or weight-for-age Z-score in the adjusted model. In a sub-analysis, including 153 children who initiated treatment during their hospital admission, and adjusting for age and sex, a doctor’s impression of TB on the day of admission was predictive of in-hospital mortality (aOR = 5.15, 95% CI 1.52–17.45, p = 0.009), while days to TB treatment initiation were not predictive of in-hospital mortality (aOR = 1.04, 95% CI 0.97–1.11, p = 0.301) (Table 5).

**Table 5:** Predictors of in-hospital mortality among children who initiated TB treatment during admission.

	Unadjusted			Adjusted^‡^ (n = 153)		
	OR	95% CI	p-value	OR	95% CI	P value
Age in years (n = 156)	1.03	0.92–1.16	0.59	0.98	0.86–1.10	0.70
Sex (n = 153)						
Female	1			1		
Male	0.44	0.18–1.10	0.078	0.39	0.15–1.02	0.054
Days to treatment initiation (n = 156)	1.01	0.94–1.08	0.79	1.04	0.97–1.11	0.30
TB suspected on admission* (n = 156)	4.02	1.30–12.47	0.016	5.15	1.52–17.45	0.009

*Abbreviations*: CI = confidence interval; OR = odds ratio; TB = tuberculosis.

^‡^Adjusted logistic regression model, including all variables in the above table.

*TB suspected on admission based on doctor’s impression listed in patient admission note.

The urban referral hospital predicted a 0.48-day increase in natural log-transformed length of stay (ln-length of stay) (95% CI 0.15–0.82, p = 0.005) in the multivariable linear regression model, but not age, sex, HIV, weight-for-age Z-score, or TBM (Table 6).

**Table 6:** Predictors of length of hospital stay* among children with tuberculosis.

	Unadjusted			Adjusted^‡^ (n = 139)		
	β^†^	95% CI	P value	β^†^	95% CI	P value
Age in years (n = 193)	0.018	-0.014–0.051	0.270	0.016	-0.022–0.055	0.404
Male sex (n = 190)	-0.140	-0.395–0.124	0.304	0.036	-0.281–0.354	0.821
Urban referral hospital (n = 193)	0.430	0.148–0.711	0.003	0.481	0.146–0.816	0.005
HIV (n = 164)	0.112	-0.168–0.392	0.432	0.125	-0.210–0.459	0.461
Weight-for-age Z-score (n = 160)	0.027	-0.031–0.085	0.354	0.036	-0.027–0.100	0.259
TB meningitis (n = 193)	0.206	-0.149–0.561	0.253	-0.128	-0.562–0.310	0.561

*Abbreviations*: β = Beta coefficient; CI = confidence interval; HIV = human immunodeficiency virus; OR = odds ratio; TB = tuberculosis.

*Length of stay was natural log transformed to best accommodate linear regression assumptions

^†^Each unit increase in predictor (e.g., age in years) predicted a (β) increase in ln-length of stay.

^‡^Adjusted linear regression model, including all variables in the above table.

In sensitivity analyses, we conducted comparisons between children with missing HIV status and those with a known HIV status. We found that children with missing HIV status were significantly younger (p<0.001) and had a shorter length of stay (p = 0.042) (data not shown). Additionally, we compared children with missing nutritional status to those with a known nutritional status. Children with missing nutritional status were more likely to be living with HIV (p = 0.036) (data not shown).

## Discussion

Our study of 201 children admitted with TB at two tertiary hospitals in Uganda, demonstrated high in-hospital mortality. Severe forms of TB, particularly TBM, were common among the hospitalised children. The odds of in-hospital mortality were significantly higher among females and three times higher among children admitted with TBM. Children with extra-pulmonary TB disease commonly presented with respiratory symptoms.

Our in-hospital mortality percentage of 17% was lower than the 32.9% previously reported in Uganda among children below 2 years of age hospitalised with presumptive TB in a rural hospital [20], where severe malnutrition and HIV-infection were the risk factors for mortality especially for children below five years of age [20]. Our mortality was also lower than the 28.4% reported in a Nigerian retrospective cohort which enrolled in-patient and out-patient children <15 years of age [21]. Conversely, it was higher than the 11% reported in a 25-year cohort of children aged 0–14 years in Peru [22], and the 1.4% in Kinshasa, Congo [23], probably due to the very low HIV prevalence in the Peru (0.1%) and Kinshasa (2.5%) cohorts, compared to the high HIV prevalence of 39% in our study.

TBM and female sex were predictive of in-hospital mortality in our cohort. TBM was also a significant risk factor of death among hospitalised children in a Nigerian retrospective cohort study. Other risk factors among the children in the Nigerian cohort included; previous TB treatment, HIV infection, extra-pulmonary TB, or a diagnosis of both pulmonary and extra pulmonary TB [21]. Our study had more children hospitalised with TBM (14%) compared to 5.7% in the Nigerian cohort, which considered both out-patient and inpatient children [21]. Extra-pulmonary TB was a significant predictor of mortality in an Ethiopian cohort of children with TB-HIV co-infection [24]. Other independent predictors of mortality among TB-HIV coinfected children in the Ethiopian cohort were anaemia, isoniazid preventive therapy initiation, and non-adherence to ART, although these were not assessed in our study [24]. TBM-related high mortality is well-documented across paediatric cohorts [25]. In a recent retrospective study in Indonesia, predictors of in-hospital mortality due to TBM included stage II disease, hydrocephalus, male sex, low-income parents, convulsions at admission, and lack of bacillus Calmette-Guérin vaccination [26].

Hospitalisations were significantly longer at the urban hospital compared with the rural hospital. This could in part be due to children with more complex and advanced disease referred to the national level, though we did not have an appropriate disease severity metric to test this assumption. Length of hospital stay, surprisingly, was not significantly influenced by age, HIV, nutritional status, or TB meningitis. Similarly, length of hospital stay did not vary by HIV status among adults hospitalised with TB in Brazil, although pulmonary TB and adverse drug reactions significantly increased hospital stay [27].

Pulmonary TB or TBM were the most common sites of disease in our cohort, aligning with paediatric TB cohorts in China [28]. Common presenting symptoms for pulmonary TB included cough, fever, and dyspnoea, while children with TBM presented with fever, convulsions, and cough. A cough in greater than half of the children presenting with TBM suggests disseminated disease, or both pulmonary and extrapulmonary disease in these children. This calls for heightened suspicion of neurologic involvement in hospitalised children with symptoms of pulmonary TB to reduce mortality and neurological sequalae due to delayed diagnosis of TBM among children with respiratory symptoms [29]. This is comparable to the 44% of children hospitalised with TBM who had chest X-ray findings suggestive of pulmonary TB and 11% with disseminated TB in a South African prospective cohort [30, 31]. The clinical presentation in our cohort resembled that of children in Nepal, where fever, cough, and weight loss, respectively, were the most common symptoms [32, 33]. In our study, despite 30% prevalence of severe malnutrition based on weight-for-age Z-score, typical symptoms of malnutrition like failure to thrive, weight loss, and poor appetite were rarely documented on admission. This observation is intriguing, especially given Uganda’s high burden of undernutrition, with stunting affecting nearly 23% of children under 5 years and about 3.6% experiencing wasting [34]. Concurrent infections in malnourished children could potentially overshadow typical TB symptoms. Moreover, the weight -for-age Z-score, which is usually a measure of wasting rather than as a measure of severe acute malnutrition, may not comprehensively capture the extent of malnutrition or correlate directly with TB symptoms in this cohort. It is also vital to consider potential misclassification bias during diagnosis of TB and SAM due to some overlap of TB and malnutrition symptoms such as weight-loss [35].

### Strengths

This study is among the few studies where there is comparison of TB treatment outcomes among hospitalised children in an urban and rural hospital within a high TB burden country. This study utilised available real-world data on HIV status of children which is a key predictor of TB treatment outcomes in children treated for TB. This study also contributes to the scarce evidence on length of hospital stay among children hospitalised with TB in African settings.

### Limitations

Convenience sampling of available patient records may have reduced variability within the selected sample. We abstracted data from paper-based hospital records which were prone to missing data on key variables such as ART regimen for HIV-infected children, TB diagnostic reports, and radiologist reports for chest X-rays done during hospitalisation. The retrospective case series limited the ability to assess for a cause-effect relationship between in-hospital mortality and identified predictors. There was limited access to follow-up data after hospital discharge to determine final TB treatment outcomes because most children who had been referred from lower health facilities, most likely returned to their nearest/referring health facility to complete TB treatment. Therefore, these results may not be generalisable to all levels of health facilities. In this study, the data on HIV status was based on reported or documented HIV status, instead of real-time HIV test results. There were no data on ART for the HIV-infected children, making it challenging to draw conclusions on associations between in-hospital mortality and ART status or ART regimen among the HIV-infected children. There was limited data on previous exposure to TB as close or household contacts, and history of using TB preventive treatment, especially among the HIV-infected children.

## Conclusions

The high in-hospital mortality among children below 15 years of age in urban and rural tertiary hospitals in Uganda, draws our attention to the possible delays in accessing TB screening, diagnosis, treatment, and prevention services among this high-risk group. Given that TBM is a risk factor of in-hospital mortality, future studies should investigate whether missed TB prevention opportunities such as BCG vaccination and TB preventive treatment are leading to these severe forms of TB disease in Ugandan children. Additionally, childhood TB diagnostic algorithms should still consider TB symptoms for clinical diagnosis, to support timely TB diagnosis towards reducing TB-associated mortality.

## Supporting information

S1 File (PDF)

## Abbreviations

ANOVA: Analysis of variance
AOR: Adjusted odds ratio
ART: Anti-retroviral therapy
ATT: Anti-TB treatment
CI: Confidence Intervals
CXR: Chest X-ray
DOT: Directly observed treatment
EPTB: Extra-pulmonary Tuberculosis
FRRH: Fort Portal Regional Referral Hospital
HIV: Human Immune-deficiency syndrome
IQR: Interquartile range
IRB: Institutional Review Board
LOS: Length of hospital stay
MAM: Moderate acute malnutrition
MHREC: Mulago Hospital Research and Ethics Committee
MNRH: Mulago National Referral Hospital
NTLP: National TB and Leprosy Program
NTRL: National Tuberculosis Reference Laboratory
OR: Odds ratio
P-BC: Bacteriologically confirmed pulmonary tuberculosis
P-CD: Clinically diagnosed pulmonary tuberculosis
PTB: Pulmonary tuberculosis
SAM: Severe acute malnutrition
SD: Standard deviation
TB: Tuberculosis
TBM: TB meningitis
WHO: World Health Organisation
ZN: Ziehl Neelsen
