# Supplementary material for: Patient characteristics and predictors of mortality among children hospitalised with tuberculosis: A six-year case series study in Uganda
Source: PLoS One. 2024 May 28;19(5):e0301107. doi: 10.1371/journal.pone.0301107 (PMC11132474; doi:10.1371/journal.pone.0301107)
Supplement: S1 File — (PDF) [file pone.0301107.s001.pdf]

Tuberculosis Risk Factors and Outcomes among Children in Urban and Rural Uganda

Protocol version and date:

V2.0; 18th January 2022

Principal Investigator: Greta Becker, Medical Student, University of Iowa, USA.

Investigators:

Ugandan Medical Student, To Be Determined, Co-Investigator: Makerere University.

Dr. Pauline Amuge, Faculty Mentor: Paediatrician, Baylor College of Medicine Children's Foundation-Uganda.

Dr. Robert Blount, Faculty Mentor: Physician-scientist, University of Iowa, USA.

Dr. Brooks Jackson, Faculty Mentor: Physician-scientist, University of Iowa, USA.

APPROVED
VAL DATE EXPIRY DATE
25 JAN 2022 24 JAN 2023
[illegible] RESEARCH ETHICS COMMITTEE
IRC

Table of Contents

Background: **4**

Objectives: **4**

METHODS **5**

- ***Study design*** *5*
- ***Study setting*** *5*

Study Procedures: **6**

Inclusion criteria: **7**

Exclusion criteria: **7**

Study Statistics: **8**

Data management: **9**

Ethical considerations: **9**

Risks **10**

Benefits **11**

- ***Participant Payment and Remuneration*** *11*

Budget **11**

INFORMED CONSENT DOCUMENT **12**

ASSENT DOCUMENT **16**

REQUEST FOR WAIVER OF INFORMED CONSENT **17**

Citations: **19**

APPROVED
[illegible]VAL DATE EXPIRY DATE
25 JAN 2022 24 JAN 2023
[illegible] RESEARCH ETHICS COMMITTEE
IRC

Abstract:

Background: The purpose of this study is to describe disease trends, outcomes, and risk factors for pediatric tuberculosis infection in rural vs urban settings.

Methods: To determine disease trends in the number of diagnosed pediatric tuberculosis cases in rural vs urban settings, in Aim 1, we will complete a retrospective study of 100 children, <15 years old, diagnosed with active tuberculosis from January 2016-June 2021 at Mulago National Referral Hospital and Fort Portal Regional Referral Hospital. We hypothesize that the number of diagnosed tuberculosis cases in both rural and urban settings will decrease over time. Using this same population, in Aim 2, we will complete a historic cohort study to determine pediatric tuberculosis outcomes in rural vs urban settings. We hypothesize that length of hospital stay is longer, and mortality is higher among children with tuberculous in rural compared to the urban hospital settings. Medical records will be reviewed using a standardized chart abstraction protocol. To identify possible risk factors for development tuberculosis infection in children, in Aim 3, we will perform a cross-sectional descriptive study of children with active tuberculosis, obtaining a risk-factor history via a questionnaire at an outpatient tuberculosis clinic.

Study Utility: These results will improve our understanding of rural healthcare disparities in Uganda and provide preliminary data for larger studies to address tuberculosis risk factors and predictors of poor tuberculosis treatment outcomes among children.

APPROVED
VAL DATE EXPIRY DATE
25 JAN 2022 24 JAN 2023
[illegible] RESEARCH ETHICS COMMITTEE
IRC

Background:

According to the World Health Organization Global Tuberculosis Report, children <15 years accounted for 12% of the 10 million tuberculosis (TB) cases and 192,000 total deaths in 2019 (1). Estimating the global burden of TB in children is difficult as many cases are undiagnosed due to the frequency of extra-pulmonary TB, non-specific symptoms, the paucibacillary nature of pediatric TB, and lower public health priority (2,3).

Uganda has a high burden of TB infection with an incidence rate of 200 per 100,000 individuals. Children <15 years account for 12% of all cases (6). In 2015, through a revised National Strategic Plan, the Uganda National Tuberculosis and Leprosy Program emphasized the importance of recognizing tuberculosis in children, including increasing the capacity of health workers to screen and diagnose childhood and extra-pulmonary TB (7).

The burden of TB disease is generally considered to be higher in urban compared to rural settings due to factors like overcrowding and higher prevalence of HIV infection (8,9,10). However, urban settings are also associated with greater healthcare delivery and improved access to healthcare providers and facilities (11). Prior observational studies have looked at pediatric TB trends and outcomes in Kampala district (12,13). However, additional research is needed to understand disease trends and outcomes in rural settings.

Objectives:

The primary objectives are:

1. To describe the trends of paediatric TB cases identified over a 5-year period among children <15 years of age in rural and urban settings.
2. To describe the TB treatment outcomes among children < 15 years of age admitted with TB disease in rural and urban settings.
3. To identify risk factors that are present in children <15 years of age with active TB in rural and urban settings.

Specific Hypotheses to be Tested:

[illegible]
VAL DATE EXPIRY DATE
25 JAN 2022 24 JAN 2023
[illegible] RESEARCH ETHICS COMMITTEE
IRC

1. The number of diagnosed TB cases initiating therapy in rural and urban settings will decrease over time.
2. Length of hospital stay in days (LOS) for children with TB is longer in the rural setting vs the urban setting.
3. Mortality is higher among children with TB in the rural hospital setting vs the urban hospital setting.

METHODS

Study design

Aims 1 and 2 are a retrospective study and historic cohort study design, respectively, collecting data on all forms of TB in the period of January 1, 2016 to June 30, 2021. Aim 3 is a descriptive cross-sectional study using a survey given to children with active TB, currently receiving treatment at an outpatient tuberculosis clinic.

Study setting

The study will be implemented at Mulago National Referral Hospital and Baylor College of Medicine Children's Foundation Uganda (Baylor-Uganda) as the urban setting, and Fort Portal Regional referral hospital as the rural setting.

Mulago National Referral Hospital is a public health facility that admits children with severe disease through the acute care unit (paediatric emergency unit) to the paediatric inpatient wards until discharge. The facility provides tertiary health care and TB diagnostic and treatment services. Children with pulmonary tuberculosis are admitted to the paediatric pulmonology-cardiology ward and children with TB meningitis are admitted to the paediatric neurology ward which are adjacent to each other. Children admitted with all forms of TB that require follow-up after discharge are discharged through the paediatric TB out-patients clinic within Mulago Hospital. Records about HIV treatment for HIV-infected children will be obtained from medical records at the HIV care clinic.

Fort Portal regional referral hospital is a regional hospital that services the entire South-Western region in Rwenzori. The facility provides tertiary health care and TB diagnostic

APPROVAL DATE EXPIRY DATE
25 JAN 2022 24 JAN 2023
RESEARCH ETHICS COMMITTEE
IRC

and treatment services. All patient records are stored and managed by the hospital records departments.

Study Procedures:

Retrospective study and historic cohort study

We will perform a retrospective chart review of electronic and written medical records for 100 children <15 years of age who were diagnosed with TB at either Mulago National Referral Hospital or a Fort Portal Regional Referral Hospital in Uganda. Cases will be included between January 1, 2016 - June 30, 2021. The medical records will be reviewed, and eligibility will be confirmed through the inclusion and exclusion criteria. If the patient is eligible, the abstractor will proceed with a standardized chart abstraction form. If the patient is not eligible, the abstractor will look for an alternative case until the 100-patient sample is reached. Data elements to be extracted include: name, medical record number, date of birth, sex, address, nationality, ethnicity, hospital admission and discharge date, weight, height, diagnosis at admission, clinical symptoms at admission, date of symptoms onset, history of prematurity, birth weight, prior medical problems, BCG vaccination, history of TB contacts, prior history of TB infection, HIV infection status, CD4 count/percentage and viral load in HIV-infected, TB diagnostics (AFB, culture, PPD), radiographic imaging reports, biopsy reports, cerebral spinal fluid labs, major procedures or interventions, TB treatment regimen, treatment adverse reactions, anti-retroviral treatment regimen for HIV-infected, final diagnosis, discharge status, and follow-up recommendations. When available, data from the 12-month follow-up visit will also be abstracted.

Descriptive cross-sectional study

We will also perform a descriptive cross-sectional study using standard questionnaire to identify possible risk factors for TB development. This questionnaire will be administered to a total of 50 children; 25 children (<15 years of age) with any form of TB disease at outpatient clinics at Mulago National Referral Hospital and 25 children (<15 years of age) with any form of TB infection at outpatient clinics at Fort Portal referral hospital. Data

VAL DATE EXPIRY DATE
2 5 JAN 2022 2 4 JAN 2023
RESEARCH ETHICS COMMITTEE
IRC

collected from the questionnaire includes length of time between symptom onset and treatment initiation, nutrition status, HIV infection, BCG status, overcrowding, exposure to indoor and outdoor air pollutants, and indirect markers of poverty. We will then look at the subject's medical record to collect information about their disease status including hospital admission date, weight, height, diagnosis codes, clinical symptoms, prematurity, birth weight, prior medical problems, TB diagnostics, radiographic imaging reports, biopsy reports, treatment regimen, culture results, and major procedures or interventions.

Inclusion criteria:

Retrospective study and historic cohort study

1. Patients presenting to Mulago National Referral Hospital or Fort Portal Regional Referral Hospital between 01-Jan-2016 to 30-June-2021
2. Males and females 0 to <15 years
3. Presumptive pulmonary or extra-pulmonary TB cases based on clinical symptoms and/or positive AFB smear, culture, or molecular-confirmed disease

Descriptive cross-sectional study

1. Males and females 0 to 15 years
2. Presumptive pulmonary or extra-pulmonary TB cases based on clinical symptoms and/or positive AFB smear, culture, or molecular-confirmed disease
3. Patients receiving outpatient TB treatment through Mulago National Referral Hospital or Fort Portal Regional Referral Hospital

Exclusion criteria:

Retrospective study and historic cohort study

1. Age greater than or equal to 15 years
2. The patient was determined not to have TB during the admission
3. Clinical notes irretrievable
4. Meningitis in post-operative setting, post-traumatic meningitis, or parameningeal infections
5. Chronic meningitis other than tuberculous meningitis

Descriptive cross-sectional study

APPROVED
VAL DATE EXPIRY DATE
25 JAN 2022 24 JAN 2023
[illegible] RESEARCH ETHICS COMMITTEE
IRC

1. Age greater than or equal to 15 years
2. Unable to contact parent or guardian

Study Statistics:

Retrospective study and historic cohort study

The primary outcome variables are TB active case trends and treatment outcome at the time of hospital discharge. We will assess all available charts of pediatric patients admitted with active TB. Means and standard deviations (SD) for continuous variables, frequency and percentage for categorical variables will be used to summarize all predictor variables and the outcome variables. Comparisons of categorical variables will be made using Chi-square and comparison of continuous variables will be completed using independent t-tests or nonparametric tests depending on normality of distribution.

To test Hypothesis 1, that the number of diagnosed TB cases initiating therapy in rural and urban settings will decrease over time, we will fit a linear regression model with number of monthly cases diagnosed as outcome and study period (in months) as the predictor with the initial month of the study period (01-Jan-2016) represented as "1" and the final month of study period 06-Jun-2021 represented as "66", accepting a linear assumption that cases are evenly distributed across each month. We will further fit an interaction term in this regression model, between dichotomous TB diagnosis location (urban or rural) and continuous study period. In an alternative model, we will test for the bivariate correlation between the annual number of cases diagnosed and the year of diagnosis by computing the Pearson's correlation coefficient, with significant temporal trend defined as a $p < 0.05$. To test Hypothesis 2, that length of hospital stay in days (LOS) for children with TBM is longer in rural vs urban settings, we will fit a linear regression model with LOS as continuous outcome and location as dichotomous predictor, controlling for a-prior selected potential confounders. To test Hypothesis 3, that mortality is higher among children with TBM in the rural hospital setting vs the urban hospital setting, we will fit a multivariable logistic regression model with in-hospital mortality as dichotomous outcome and location as dichotomous predictor.

APPROVED
VAL. DATE 25 JAN 2022
EXPIRY DATE 24 JAN 2023
RESEARCH ETHICS COMMITTEE
IRC

Sample size calculations to determine the appropriate number of retrospective charts to review are based on Hypothesis 2 using the t-statistic as calculated at quesgen.com. We will test associations between hospital setting and outcomes (LOS and mortality) in the entire cohort of children with active tuberculosis. Testing our Hypothesis 2, n=100, we are adequately powered at > 80% to detect differences in LOS outcome of > 16 days, an adequate effect size as LOS for children with active TB is typically several weeks (median 36 days, IQR 21-60 days in the (Blount 2014) cohort.

Descriptive cross-sectional study

Rural vs. Urban baseline characteristics will be compared using the Chi-square Fisher exact test for categorical variables and the t-test for continuous variables.

Data management:

Data will be recorded on standardized paper chart abstraction forms and within one week entered into the REDCap cloud-based password-protected database, with preset validation tools to ensure the accuracy of the data entry. After data abstraction and administration of the questionnaire, the medical record number will be converted to a unique study number for each participant. A master data sheet will include medical record numbers with corresponding UID number. A separate data sheet used for statistical analyses will have only the UID numbers. Data will be stored on password-protected files. A copy of the password-protected filed will be stored on one of the servers provided by the Office of Research. The data will be deleted within seven years following publishing.

Ethical considerations:

The project will be approved by the University of Iowa, Mulago Hospital Institutional Review Board (IRB), and Uganda National Council for Science and Technology. All data collected and generated during this study will remain confidential in accordance with HIPAA and institutional policies. The collected data will only be used to accomplish the outlined study objectives and for no other purposes.

Retrospective study and historic cohort study

APPROVED
APPROVAL DATE EXPIRY DATE
25 JAN 2022 24 JAN 2023
RESEARCH ETHICS COMMITTEE
IRC

We are requesting a waiver of participant assent and a waiver of parental consent for the chart abstraction based on the following qualifications: the research is limited to existing data; the data involves minimal risk to the subject; the waiver would not adversely affect the rights of the subjects.

Descriptive cross-sectional study

Age-appropriate assent and consent forms in Lugandan will be prepared for the risk-factor questionnaires.

Risks:

The study is a minimal risk study. The primary risks are breach of confidentiality, and some questions may be uncomfortable to answer. A breach of confidentiality violates a subject's rights and risks harm to the subject and family ranging from social stigmatization, damage to social status, damage to economic status, including loss of employment or other benefits. Uncomfortable questions could cause unwanted psychological or emotional stress.

The consequences of a breach of confidentiality include dealing with ramifications of lawsuits, loss of professional relationships, and employee termination. Data in paper format will be stored in a locked filing cabinet in the locked office of the research team. Computer data will be stored in a password-protected cloud-based REDCap database. The patient identifiers including medical record number and birthdate will be converted to a unique study number and age. Unanticipated problems and risks to subjects will be monitored throughout the study.

We will do our best to avoid asking sensitive questions. For sensitive topics like socioeconomic status, we will ask questions indirectly by asking about home ownership, phone ownership, insurance status, and education. Staff pediatricians will refer the subject for psychological counseling if necessary.

APPROVED
APPROVAL DATE EXPIRY DATE
25 JAN 2022 24 JAN 2023
[illegible] RESEARCH ETHICS COMMITTEE
IRC

If any unanticipated problems related to the research involving risks to subjects happen during the project, these risks and problems will be reported to the IRBs. Unanticipated problems that do not involve risks to the subjects will be submitted to the IRB at the time of continuing review.

Benefits:

There are no direct benefits to children in this study. However, there are potential benefits to children who are at risk for developing tuberculosis infection by improving the understanding of rural healthcare disparities in Uganda.

Participant Payment and Remuneration*:*

Participants completing the risk factor questionnaire will be compensated with $8.40 (Ugx 30,000), for their time, effort and inconveniences during the study.

Budget:

<u>Procedures:</u>

EMR interrogation fee, $150

Translation of consents to local language, $6 (Ugx 20,000) per page, $24

<u>Personnel:</u>

Makerere University medical student, $200

Records Assistant Mulago Hospital, $450

Data entry officer Mulago Hospital, $450

Records Assistant Fort Portal Hospital, $450

Data entry officer Fort Portal Hospital, $450

APPROVED
[illegible]VAL DATE EXPIRY DATE
25 JAN 2022 24 JAN 2023
[illegible] RESEARCH ETHICS COMMITTEE
IRC

<u>Administrative fees:</u>

Initial IRB application, $400

Office supplies for chart abstraction form/consent forms/questionnaires, $50

INFORMED CONSENT DOCUMENT

Project Title: **Tuberculosis Risk Factors and Outcomes among Children in Urban and Rural Uganda**

Principal Investigator: Greta Becker, medical student
Faculty Mentor(s): Dr. Pauline Amuge, paediatrician
Dr. Robert Blount, physician-scientist

Research Team Contact: Greta Becker: (563)-552-9422 (cell); greta-becker@uiowa.edu

- If you are the parent/guardian of a child under 18 years old who is being invited to be in this study, the word "you" in this document refers to your child. You will be asked to read and sign this document to give permission for you and your child to participate.
- If you are a teenager reading this document because you are being invited to be in this study, the word "you" in this document refers to you. You will be asked to read and sign this document to indicate your willingness to participate.

This consent form describes the research study to help you decide if you want to participate. This form provides important information about what you and your parent will be asked to do during the study, about the risks and benefits of the study, and about your rights as a research subject.

- If you have any questions about or do not understand something in this form, you should ask the research team for more information.
- You should discuss your participation with anyone you choose such as family or friends.
- Do not agree to participate in this study unless the research team has answered your questions and you decide that you want to be part of this study.

WHAT IS THE PURPOSE OF THIS STUDY? This is a research study. We are inviting you and your parent to participate in this research study because you have been diagnosed and hospitalized with tuberculosis.
The purpose of this research study is to describe the risk factors for development of severe tuberculosis among children in Uganda.

HOW MANY PEOPLE WILL PARTICIPATE? Approximately **50** people and a parent/guardian will take part in this study conducted by investigators at the University of Iowa.

HOW LONG WILL MY PARENT AND I BE IN THIS STUDY? If you agree to take part in this study, your involvement will last for one visit. All procedures will take approximately 30 minutes to complete.

APPROVED
VAL. DATE EXPIRY DATE
25 JAN 2022 24 JAN 2023
RESEARCH ETHICS COMMITTEE
IRC

WHAT WILL HAPPEN DURING THIS STUDY?

- We will ask you and your parent some questions about your living situation, environmental exposures, and health. You are free to skip any questions that you wish not to answer. Again, parents may answer for minor children participating.
- We will look at your medical record to collect information about your current hospitalization.

WHAT ARE THE RISKS OF THIS STUDY?

You may experience one or more of the following risks indicated below from being in this study. In addition to these, there may be other unknown risks, or risks that we did not anticipate, associated with being in this study.

- Some questions may be uncomfortable to answer.
- There is a risk of loss of confidentiality of data.

Your information collected as part of the research, even if identifiers are removed, will not be used or distributed for future research studies.

WHAT ARE THE BENEFITS OF THIS STUDY?

You will not directly benefit from being in this study. However, this research may improve our understanding of tuberculosis risk factors and predictors of poor tuberculosis treatment outcomes which may help other children in the future.

WILL IT COST ME ANYTHING TO BE IN THIS STUDY? You will not have any costs for participating in this research study.

WILL I BE PAID FOR PARTICIPATING?

Participants completing the risk factor questionnaire will be compensated Shs. 30,000 ($8.4) for their time, effort and inconveniences during the study.

WHO IS FUNDING THIS STUDY?

Principal Investigator Personal Funds

WHAT ABOUT CONFIDENTIALITY? We will keep your participation in this research study confidential to the extent permitted by law. However, it is possible that other people such as those indicated below may become aware of your participation in this study and may inspect and copy records pertaining to this research. Some of these records could contain information that personally identifies you.

- Federal government regulatory agencies,
- Makerere University School of Medicine Research and Ethics committee
- Mulago Hospital Institutional Review Board
- Auditing departments of the University of Iowa, and
- The University of Iowa Institutional Review Board (a committee that reviews and approves research studies)

To help protect your confidentiality, we will complete our study in a private setting and keep your personal information on a locked computer. Data in paper format will be

APPROVAL [illegible]
[illegible]VAL DATE [illegible]XPIRY DATE
25 JAN 2022 24 JAN 2023
[illegible] RESEARCH ETHICS COMMITTEE
IRC

stored in a locked filing cabinet in the locked office of the research team. Computer data will be stored in a password-protected cloud-based database. The patient identifiers including medical record number and birthdate will be converted to a unique study number and age. If we write a report or article about this study or share the study data set with others, we will do so in such a way that you cannot be directly identified.

IS BEING IN THIS STUDY VOLUNTARY? Taking part in this research study is completely voluntary. You may choose not to take part at all. If you decide to be in this study, you may stop participating at any time. If you decide not to be in this study, or if you stop participating at any time, you won't be penalized or lose any benefits for which you otherwise qualify.

Will I Receive New Information About the Study while Participating? If we obtain any new information during this study that might affect your willingness to continue participating in the study, we'll promptly provide you with that information.

WHAT IF I HAVE QUESTIONS? We encourage you to ask questions. If you have any questions about the research study itself, please contact: Greta Becker at +1 563-552-9422, email greta-becker@uiowa.edu or Dr. Pauline Amuge at +256 782932508, email pamuge@baylor-uganda.org. If you experience a research-related injury, please contact: Dr. Pauline Amuge at +256 782932508, or Dr. Robert Blount at (319)-384-1107.

If you have questions, concerns, or complaints about your rights as a research subject or about research related injury, please contact the University of Iowa Human Subjects Office, 105 Hardin Library for the Health Sciences, 600 Newton Rd, The University of Iowa, Iowa City, IA 52242-1098, (319) 335-6564, or e-mail irb@uiowa.edu or Dr. Fred Nakwagala, chairperson of Mulago Hospital Institutional Review Board at +256 772 325869 or Ponsiano Ocama, chairperson of the Makerere University School of Medicine Research and Ethics Committee (SOM REC) at 0414-533541 or 0772421190. To offer input about your experiences as a research subject or to speak to someone other than the research staff, call The University of Iowa Human Subjects Office at the number above.

This Informed Consent Document is not a contract. It is a written explanation of what will happen during the study if you decide to participate. You are not waiving any legal rights by signing this Informed Consent Document. Your signature indicates that this research study has been explained to you, that your questions have been answered, and that you agree to take part in this study. You will receive a copy of this form.

Child/Subject's Name (printed):

____

APPROVED
VAL DATE 25 JAN 2022
EXPIRY DATE 24 JAN 2023
RESEARCH ETHICS COMMITTEE
IRC

(Signature of Child/Subject) (Date)

Parent/Subject's Name (printed):

(Signature of Parent/Subject) (Date)

Parent/Guardian's Name and Relationship to Child/Subject:

(Name - printed) (Relationship to Child/Subject - printed)

(Signature of Parent/Guardian for child/subject's participation) (Date)

Statement of Person Who Obtained Consent

I have discussed the above points with the subject or, where appropriate, with the subject's legally authorized representative. It is my opinion that the subject understands the risks, benefits, and procedures involved with participation in this research study.

(Signature of Person who Obtained Consent) (Date)

APPROVED
[illegible]VAL DATE [illegible]XPIRY DATE
25 JAN 2022 24 JAN 2023
[illegible] RESEARCH ETHICS COMMITTEE
IRC

ASSENT DOCUMENT

Project Title: **Tuberculosis Risk Factors and Outcomes among Children in Urban and Rural Uganda**

Investigator(s): **Greta Becker, BS**

We are doing a research study. A research study is a special way to find out about something. We are trying to learn why children develop tuberculosis.

If you decide that you want to be in this study, this is what will happen: we will ask you some questions about your life outside of the hospital and we will look at your medical record to learn about your health.

Answering the questions might make you uncomfortable, but you don't have to answer if you don't want to.

We don't know if being in this research study will help you. But we hope to learn something that will help other people someday.

When we are done with the study, we will write a report about what we have found out. We won't use your name in the report.

You don't have to be in this study. It's up to you. If you say okay now, but you change your mind later, that's okay too. All you have to do is tell us.

If you want to be in this study, please tell me now.

____________________________________, has verbally agreed to participate in the study

(Child participant's name)

____________________________________ __________________

(Signature of person obtaining assent) (Date)

____

(Name of person obtaining assent)

APPROVED
[illegible]VAL DATE EXPIRY DATE
25 JAN 2022 24 JAN 2023
[illegible] RESEARCH ETHICS COMMITTEE
IRC

REQUEST FOR WAIVER OF INFORMED CONSENT

Project Title: **Tuberculosis Risk Factors and Outcomes among Children in Urban and Rural Uganda**

Principal Investigator: Greta Becker, medical student
Faculty Mentor(s): Dr. Pauline Amuge, paediatrician
Dr. Robert Blount, physician-scientist

Research Team Contact: Greta Becker: (563)-552-9422 (cell); greta-becker@uiowa.edu

The researcher plans to determine the prevalence of tuberculous meningitis and discharge outcomes in rural vs urban hospitals in Uganda.

The proposed research plan is to:
Complete a retrospective case series study of 100 children, <15 years old, diagnosed with active tuberculosis from January 1, 2016- 30th June 2021. Medical records will be reviewed using a standardized chart abstraction protocol.

Data to be collected:
Name, medical record number, date of birth, sex, address, nationality, ethnicity, hospital admission and discharge date, weight, height, diagnosis at admission, clinical symptoms at admission, date of symptoms onset, history of prematurity, birth weight, prior medical problems, BCG vaccination, history of TB contacts, prior history of TB infection, HIV infection status, CD4 count/percentage and viral load in HIV-infected, radiographic imaging reports, biopsy reports, cerebral spinal fluid labs, culture results, major procedures or interventions, TB treatment regimen, treatment adverse reactions, anti-retroviral treatment regimen for HIV-infected, final diagnosis, discharge status, and follow-up recommendations. When available, data from the 12-month follow-up visit will also be abstracted.

Is there sufficient justification for IRB to approve a waiver of informed consent?

1. The research involves minimal risk. The probability and magnitude of harm or discomfort in the research project are not greater than those encountered in daily life. This is a non-interventional, observational study. Some of the data abstracted from the chart is sensitive information. There is a potential risk of breach of privacy and confidentiality. We will complete our study in a private setting and keep personal information on a locked computer. Data in paper format will be stored in a locked filing cabinet in the locked office of the research team. Computer data will be stored in a password-protected cloud-based REDCap database. The patient identifiers, including medical record number and birthdate, will be converted to a unique study number and

APPROVED
APPROVAL DATE EXPIRY DATE
25 JAN 2022 24 JAN 2023
[illegible] RESEARCH ETHICS COMMITTEE
IRC

age. If we write a report or article about this study or share the study data set with others, we will do so in such a way that the subject cannot be directly identified.

2. The rights and welfare of the individuals would not be adversely affected. This study will only involve the collection of existing data and measures will be taken to protect the privacy and confidentiality of subjects, as described above. For these reasons, the waiver of consent will not adversely affect the rights and welfare of the study subjects.

3. The research could not practicably be carried out without a waiver. Identifying and contacting 100 potential subjects would be difficult in the one-month time frame allotted for this project. Additionally, potential subjects may be deceased or lost to follow-up and the data being reviewed does not contain accurate contact information for the potential subjects.

4. It would not be appropriate to provide these subjects with information about the results of the research. The results of this study will have no effect on the subject. There is no anticipated benefit to the subjects that would change what has already occurred.

APPROVED
[illegible]VAL DATE EXPIRY DATE
25 JAN 2022 24 JAN 2023
[illegible] RESEARCH ETHICS COMMITTEE
IRC

Citations:

(1) Global tuberculosis report 2019. Geneva: World Health Organization; 2019. Licence: CC BY-NC-SA 3.0 IGO.

(2) Dodd, P. J., Gardiner, E., Coghlan, R., & Seddon, J. A. (2014). Burden of childhood tuberculosis in 22 high-burden countries: a mathematical modelling study. *The Lancet. Global health*, *2*(8), e453–e459.

(3) Nelson, L. J., & Wells, C. D. (2004). Global epidemiology of childhood tuberculosis. *The international journal of tuberculosis and lung disease: the official journal of the International Union against Tuberculosis and Lung Disease*, *8*(5), 636–647.

(4) Thwaites, G. E., van Toorn, R., & Schoeman, J. (2013). Tuberculous meningitis: more questions, still too few answers. *The Lancet. Neurology*, *12*(10), 999–1010.

(5) Chiang, S. S., Khan, F. A., Milstein, M. B., Tolman, A. W., Benedetti, A., Starke, J. R., & Becerra, M. C. (2014). Treatment outcomes of childhood tuberculous meningitis: a systematic review and meta-analysis. *The Lancet. Infectious diseases*, *14*(10), 947–957.

(6) Uganda National TB and Leprosy Program (2020). Republic of Uganda Ministry of Health. https://www.health.go.ug/cause/uganda-national-tb-and-leprosy-program-july-2019-june-2020-report/

(7) Uganda Tuberculosis Roadmap Overview, Fiscal Year 2021. USAID; 2021.

(8) World Health Organization. Global tuberculosis report 2014. Available: https://www.who.int/tb/publications/global_report/gtbr14_main_text.pdf

(9) Datiko, D. G., Yassin, M. A., Chekol, L. T., Kabeto, L. E., & Lindtjørn, B. (2008). The rate of TB-HIV co-infection depends on the prevalence of HIV infection in a community. *BMC public health*, *8*, 266. https://doi.org/10.1186/1471-2458-8-266

(10) Mutembo, S., Mutanga, J. N., Musokotwane, K., Kanene, C., Dobbin, K., Yao, X., Li, C., Marconi, V. C., & Whalen, C. C. (2019). Urban-rural disparities in treatment outcomes among recurrent TB cases in Southern Province, Zambia. *BMC infectious diseases*, *19*(1), 1087. https://doi.org/10.1186/s12879-019-4709-5

(11) Abubakar, I., Crofts, J. P., Gelb, D., Story, A., Andrews, N., & Watson, J. M. (2008). Investigating urban-rural disparities in tuberculosis treatment outcome in England and Wales. *Epidemiology and infection*, *136*(1), 122–127. https://doi.org/10.1017/S0950268807008333

(12) Wobudeya, E., Lukoye, D., Lubega, I. R., Mugabe, F., Sekadde, M., & Musoke, P. (2015). Epidemiology of tuberculosis in children in Kampala district, Uganda, 2009-

APPROVED
VAL DATE EXPIRY DATE
25 JAN 2022 24 JAN 2023
[illegible] RESEARCH ETHICS COMMITTEE
IRC

2010; a retrospective cross-sectional study. *BMC public health*, *15*, 967. https://doi.org/10.1186/s12889-015-2312-2

(13) Wobudeya, E., Sekadde-Kasirye, M., Kimuli, D., Mugabe, F., & Lukoye, D. (2017). Trend and outcome of notified children with tuberculosis during 2011-2015 in Kampala, Uganda. *BMC public health*, *17*(1), 963. https://doi.org/10.1186/s12889-017-4988-y

(14) Blount, R. J., L. G. Jarlsberg, K. R. Daly, et al. (2012). "Serologic responses to recombinant Pneumocystis jirovecii major surface glycoprotein among Ugandan patients with respiratory symptoms." PLoS One 7(12): e51545. PMCID: 3528778. DOI: 10.1371/journal.pone.0051545

(15) Blount, R. J., B. Tran, L. G. Jarlsberg, et al. (2014). "Childhood tuberculosis in northern viet nam: a review of 103 cases." PLoS One 9(5): e97267. PMCID: PMC4018290. DOI: 10.1371/journal.pone.0097267

APPROVED
APPROVAL DATE EXPIRY DATE
25 JAN 2022 24 JAN 2023
MULAGO RESEARCH ETHICS COMMITTEE
IRC
